# Cities as hotspots of indirect water consumption: The case study of Hong Kong

**DOI:** 10.1016/j.jhydrol.2017.12.004

**Published:** 2019-06

**Authors:** D. Vanham, B.M. Gawlik, G. Bidoglio

**Affiliations:** European Commission, Joint Research Centre, Directorate for Sustainable Resources, Via E. Fermi 2749, 21027 Ispra, VA, Italy

## Abstract

During the last years, the city of Hong Kong has made large investments to make its urban water supply system more water efficient and sustainable. As such, its municipal water abstraction – often defined as direct water use – has decreased from 355 litre per capita per day (l/cap/d) in 2005 to 326 l/cap/d in 2013. Due to its political history, Hong Kong is unique in the world in data availability on urban food consumption. It is therefore the ideal case study to show typical urban food consumption behaviour and its related indirect water use. The objective of this paper is to show the large water quantities associated with indirect water use and that the citizens of Hong Kong can additionally save much more water by looking at this indirect water use. The current average diet in Hong Kong is very different to the average Chinese diet. It is characterised by a high intake of water intensive products like animal products and sugar, leading to a food related indirect water use or water footprint (WF_cons_) of 4727 l/cap/d. According to recommendations from the Chinese Nutrition Society for a healthy diet, the intake of some product groups should be increased (vegetables and fruit) and of other product groups reduced (sugar, crop oils, meat and animal fats). This would result in a reduction of the WF_cons_ of 40% to 2852 l/cap/d. Especially the reduced intake of meat (including offals) from currently 126 kg per capita per year (kg/cap/yr) to the recommended value 27 kg/cap/yrwould results in a substantial WF_cons_ reduction. Meat consumption in Hong Kong is extremely high. A pesco-vegetarian diet would result in a reduction of 49% (to 2398 l/cap/d) and a vegetarian diet in a 53% (to 2224 l/cap/d) reduction. Hong Kong citizens can thus save a lot of water through a change in their diet. Many of the products consumed, contribute to different levels of blue water scarcity in the regions of origin Hong Kong imports from. This poses a water-related risk to food security in Hong Kong. As all diet scenarios also result in a lower blue WF_cons_, they decrease this risk. In order to become sustainable, (mega)cities should reduce their dependency on distant resources and ecosystems.

## Highlights

• Hong Kong’s municipal water footprint (WF) is 326 l/cap/d.
• Its indirect water use (or WF) due to food consumption is 4727 l/cap/d.
• Hong Kong has a typical urban diet, with too high animal product consumption.
• Some products consumed in Hong Kong contribute to blue water scarcity.
• Citizens of Hong Kong can reduce water resource use by changing their diets.

## Graphical abstract

**Figure:**
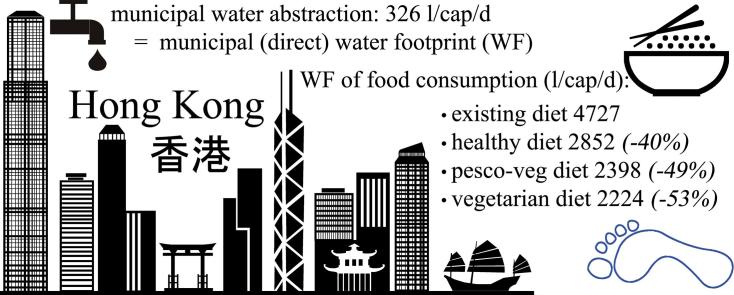

## 1 Introduction

Cities are hotspots of consumption. The UN has identified sustainable cities and communities as one of its Sustainable Development Goals (SDGs). As such, the extent to which urban dwellers consume resources is key on the path to a sustainable world. One of these resources is water, which is consumed in a direct and indirect way by city inhabitants (Vanham and Bidoglio, 2014). While most people are aware of their direct water consumption, they are not of their indirect water consumption. The latter is defined as the water that is required to produce the goods and services city inhabitants consume, i.e. their water footprint (Hoekstra and Mekonnen, 2012).

Many modern cities have invested a lot of money in a more water efficient and sustainable urban water cycle, e.g. by reducing water leakages (Koop and van Leeuwen, 2015). As such, direct urban water use has decreased in the last decade in many modern cities. Awareness raising campaigns and policy have generally focused on increasing water efficiency in domestic and industrial water use, e.g. by using tap water wisely or by increasing water efficiency of washing machines or dishwashers. The Hong Kong Special Administration Region (HKSAR) government has during recent years implemented a wide range of water conservation measures, which include metering and a tiered water tariff, large-scale replacement and rehabilitation of ageing pipelines to reduce leakage, the use of sea water for toilet flushing and education and publicity programmes (Hong Kong Statistics, 2015b, Yue and Tang, 2011).

Although these measures are very important to reduce direct water use, the objective of this paper is to show that the citizens of Hong Kong can additionally save much more water by looking at their indirect water use. Urban centres are very much dependent on distant resources, ecosystems and populations. More particularly, our objective is to show that – using Hong Kong as a case study – 1) indirect water use is responsible for enormous water quantities in cities and 2) water can also be saved by urban dwellers by looking at their diets.

Indeed, based upon planetary boundaries (Rockstrom et al., 2009), sustainable solutions in water management need to come from both the supply and demand side, within a systems approach (EXPO EU Scientific Committee, 2015), addressing the water-energy-food-ecosystems nexus context (Vanham, 2016). Supply-side solutions include water efficiency in the urban water cycle or agricultural water production (Godfray et al., 2010), whereas demand-side solutions include diet habits (Godfray et al., 2010, Vanham et al., 2013b) or the reduction of food waste (Vanham et al., 2015). Although cities can never become fully self-sufficient, it is important that they contribute to global sustainability and resilience by optimizing resource use, increasing efficiency and minimizing waste (Elmqvist, 2014).

In this paper, we analyse the indirect water consumption related to food intake in the city of Hong Kong, by means of the water footprint concept (Hoekstra and Mekonnen, 2012, Vanham and Bidoglio, 2013). Both direct water use and indirect water use together are defined as the water footprint of consumption (WF_cons_) of a city. Water footprint assessments on the city level have not been the focus of research in the past (Paterson et al., 2015). During recent years several studies have however been conducted, e.g. Hoff et al., 2014, Jenerette et al., 2006, Kennedy et al., 2015, Ma et al., 2015, Rushforth and Ruddell, 2016. City water footprint analyses related to food consumption including diet scenarios have been conducted for a range of pan-European cities, i.e. for Milan (Vanham and Bidoglio, 2014), Dutch cities (Vanham et al., 2016b), Mediterranean cities (Vanham et al., 2016a, Vanham, 2016) and Nordic cities (Vanham et al., 2017).

In our study we focus on indirect water use related to food consumption in Hong Kong. Today overconsumption and undernutrition go together in China. There are now important differences in food consumption behaviour between urban and rural regions in China, not at least related to increased income differences (Dong and Hu, 2010, Zhang et al., 2008). Also in Hong Kong overconsumption and undernutrition go hand in hand, with associated health problems. A total of 47% of the Hong Kong population (54% of the males and 41% of the females) are overweight or obese according to the World Health Organisation (WHO) proposed classification of weight by BMI in adult Asians (CUHK, 2010). Another 9% is underweight (CUHK, 2010).

We assess the WF_cons_ of food for:

• The current situation (reference period 1996–2005 or REF)
• Three diet scenarios:
o a healthy diet as recommended by the Chinese food guide pagoda (Chinese Nutrition Society, 2015) or HEALTHY
o a healthy pesco-vegetarian diet or PESCO-VEG
o a healthy vegetarian diet or VEG

The production of certain agricultural products (crop and livestock products) can attribute to blue water scarcity. Hong Kong might import food items from regions where water scarcity occurs, thereby putting a risk to its food security. Therefore, we make a general evaluation of whether products imported by Hong Kong are produced under blue water scarcity conditions, the latter being the ratio between consumptive water use and ecologically available water.

## 2 Methodology

### 2.1 Accounting framework

**Fig. 1:**
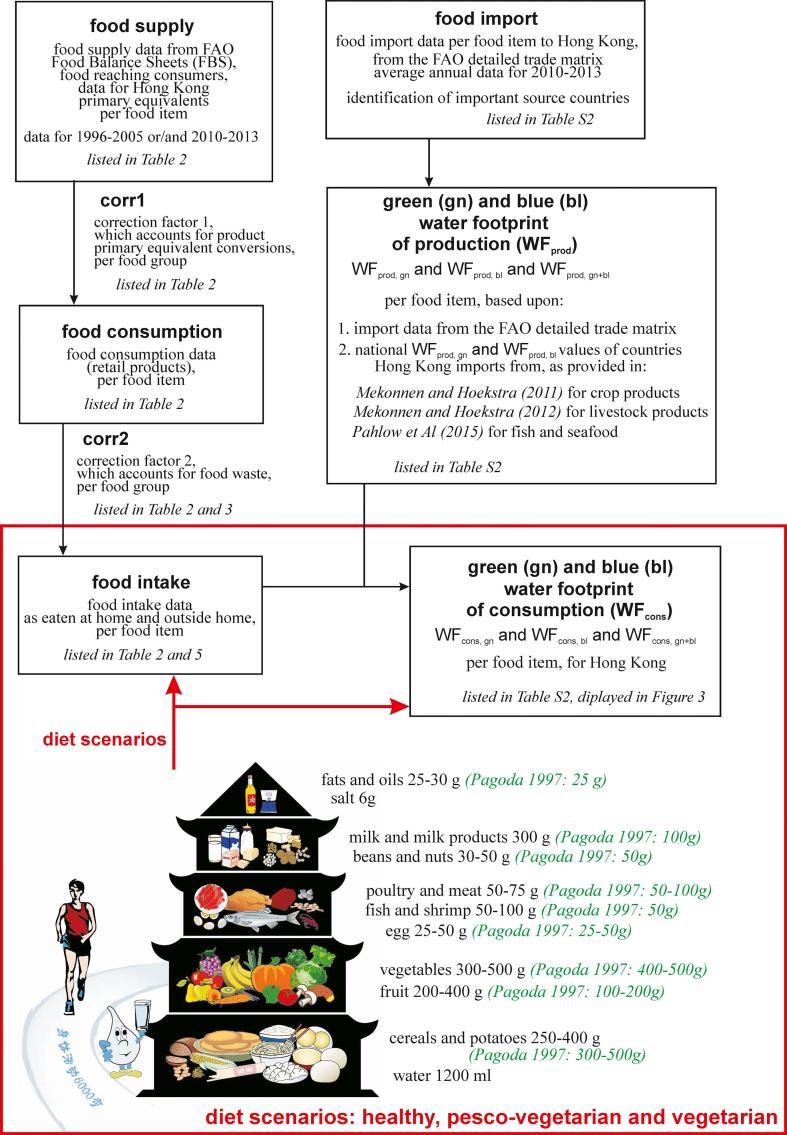
Workflow scheme of the methodology used in this study. Diet scenarios are based upon the Chinese dietary guidelines (Chinese Nutrition Society, 2015), as displayed in the Chinese food guide pagoda (Values per capita per day).

A list of abbreviations used in this study is given in Table 1. A workflow scheme of the methodology used in this study is displayed in Fig. 1.

**Table 1:** Abbreviations used in this study.

Abbreviation	Definition
SDGs	Sustainable Development Goals
HKSAR	Hong Kong Special Administration Region
WHO	World health Organisation
FAO	Food and Agricultural Organisation of the United Nations
FBS	Food balance Sheets
WF	Water footprint
WFN	Water Footprint Network
WF_prod_	Water footprint of production
WF_cons_	Water footprint of consumption
gn	green (e.g. as in WF_cons, gn_)
bl	blue (e.g. as in WF_cons, bl_)
gn + bl	green + blue (e.g. as in WF_cons, gn+bl_)
WS	Water stress
REF	The reference period, 1996–2005
HEALTHY	Healthy diet scenario
PESCO-VEG	Pesco-vegetarian diet scenario
VEG	Vegetarian diet scenario
FBDG	Food based dietary recommendations
l/cap/d	Litres per capita per day
EFR	Environmental flow requirement

To assess WF values, the volumetric approach of the Water Footprint Network or WFN (Hoekstra et al., 2011, Hoekstra and Mekonnen, 2012) is applied. We use the blue and green WF components. Following the definition of Rockström et al. (2009), green water is the soil water held in the unsaturated zone, formed by precipitation and available to plants. Blue water refers to liquid water in rivers, lakes, wetlands and aquifers. Irrigated agriculture receives blue water (from irrigation) as well as green water (from precipitation), while rainfed agriculture receives only green water. The green WF is thus the rainwater consumed by crops. The inclusion of green water is a necessity in integrated water resources management (IWRM), as argued by most authors and institutions working on IWRM (Gerten et al., 2013, Hoekstra, 2016, Hoff et al., 2014, Jalava et al., 2016, Jalava et al., 2014, Karimi et al., 2013, Miina et al., 2016, Ran et al., 2016, Rockström et al., 2014, Schyns et al., 2015, Vanham, 2012). We do not use the grey WF, as its quantification is very dependent on data availability (Thaler et al., 2012, Vanham and Bidoglio, 2013).

To compute the water footprint of consumption (WF_cons_) related to food consumption, we use FAO Food Balance Sheets (FBS) for Hong Kong for the reference period 1996–2005 and/or 2010–2013. As indicated, these data are unique as they provide for food consumption behaviour of a city. When average annual food consumption data of a food item during 2010–2013 differed more than 5% from the 1996–2005 values, we used 2010–2013 data (with 2013 being the latest data available during the writing of this paper). Otherwise we used 1996–2005 data. To compute WF_cons_ values for Hong Kong, we need WF of production (WF_prod_) values for the food product (groups) consumed in Hong Kong. Almost 100% of this food is imported. Therefore, we use trade data per food item from the FAO detailed trade matrix, more specifically average annual values for the period 2010–2013. By using 4 years, we account for interannual fluctuations. We select countries so that they represent at least 50% of total food item imports. For these countries, we obtain average national green and blue WF_prod_ values from (Mekonnen and Hoekstra, 2011) for crop products and from (Mekonnen and Hoekstra, 2012) for livestock products. For the remaining food item import (because it is practically almost impossible to account for all imports because some are very minor in quantity), we use average global WF_prod_ values. To compute the green and blue WF_cons_ of a food item consumed in Hong Kong, we weigh the WF_prod_ values of origin countries according to their contribution to the total import amounts. All these values (food item FAO FBS values, main countries of import origin with WF_prod_ values, food item WF_cons_ values for Hong Kong) are displayed in Table S2.

We also include a WF for fish and seafood, because a recent publication (Pahlow et al., 2015) has provided relevant WF quantifications for fish feed. For pond evaporation, the blue WF value of Verdegem et al. (2006) is taken. Aquaculture will soon surpass wild fisheries as the main source of seafood. This reflects the transition, which happened on land in the past with the evolution from hunting to farming. Neglecting the WF of fish and seafood consumption therefore underestimates the WF_cons_ related to food consumption.

The FAO FBS values for Hong Kong are data on food supply (displayed as “food supply data” in Table 2), i.e. food reaching the consumer in private households, as well as that in the non-household sector, i.e. catering establishments, schools, hospitals, prisons, armed forces' bases and other communities. The data are given on an “as purchased” basis, i.e. as the food leaves the retail shop or enters the household by other means.

**Table 2:** Food supply, food consumption and food intake amounts for Hong Kong for the different product groups, reference period.

	Food supply (kg/cap/yr)	corr1	Food consumption (retail product)	corr2	Food intake
Cereals	106.3		94.3	0.9	84.9
of which wheat	49.5	0.8^a^	39.6	0.9	35.6
of which rice	51.0	1	51.0	0.9	45.9
of which others	5.8	0.63–0.75^a^	3.8	0.9	3.4
Starchy roots	25.2		25.2	0.9	22.7
of which potatoes	23.8	1	23.8	0.9	0.8
of which others	0.9	1^a^	0.9	0.9	21.5
Sugar	36.9	1	36.9	0.95	34.9
Crop oils	11.0	1	11.0	0.95	10.5
Vegetables	111.9	1	111.9	0.9	100.7
Fruit	87.1	1	87.1	0.94	81.9
Pulses, nuts and oilcrops	20.4	1	20.4	0.95	19.3
Meat	154.2		110.4	0.93	102.7
of which pork	62.6	0.7^b^	43.8	0.93	40.8
of which beef	25.1	0.8^b^	20.1	0.93	18.7
of which poultry	61.4	0.7^b^	43.0	0.93	40.0
of which other meat	5.1	0.7^b^	3.5	0.93	3.3
Offals edible	24.5	1	24.5	0.93	22.8
Fish and seafood	62.9	0.5^c^	31.4	0.92	28.9
of which fish	36.4	0.5^c^	18.2	0.92	16.7
of which shellfish	27.7	0.5^c^	13.6	0.92	12.5
Animal fats	6.6	1	6.6	0.95	6.3
Milk and milk products	95.2	1^d^	95.2	0.95	90.5
Eggs	14.0	1	14.0	0.95	13.3
Stimulants	6.3	1	6.3	0.95	6.0
Spices	1.0	1	1.0	0.95	0.9
Alcoholic Beverages	30.0	1	30.0	0.95	28.5
**SUM**	**793.5**		**706.4**		**654.8**

a Values based on (FAO, 1972, Vanham et al., 2013a, Westhoek, et al., 2011).

b Source: for pork and beef (Hong Kong Statistics, 2003); for poultry and other meat (Westhoek et al., 2011).

c Source (FAO, 1989) – http://www.fao.org/docrep/003/t0219e/t0219e01.htm.

d The value 1 is valid for milk, yoghurt and cream. The conversion factor for cheese is different from 1 (Vanham et al., 2013a), but as cheese is consumed in neglectable quantities in Hong Kong, this is not taken into account.

Quantities are provided on the basis of “primary equivalents”. Within the FAO FBS, food data are standardized in that processed commodities are converted back to their “primary equivalent”. This is for standardization (different countries report their data to the FAO), simplification and limitation of the number of commodities within the FBS. E.g., instead of listing flour of wheat, bread or pasta separately in the FBS, they are quantified as wheat equivalent. Similarly, meat is quantified as carcass weight in the FBS. Fish and seafood are expressed as live weight equivalents.

In order to compute food intake amounts (food quantities people actually eat) based upon FAOSTAT FBS food supply amounts, two correction factors are necessary. With the first one (corr1), food consumption (retail product) amounts are computed from food supply amounts. The second one (corr2) accounts for consumer food waste (both at home and at the food service/catering level) and computes food intake amounts from food consumption (retail product) amounts. For all product groups these values are listed in Table 2. This methodology was also used and described in Vanham et al., 2013a, Vanham et al., 2013b). Product consumer food waste values for Hong Kong are not available. As such, other sources were used to determine corr2 values, as listed in Table 3.

**Table 3:** Literature and chosen values for food waste at the consumer level for the different product groups. With HH CH = households China; CA BEI = Catering in Beijing; CA LHA = Catering in Lhasa; CO CH = total consumption China (households and catering); CO EU = total consumption EU (households and catering).

	Literature values on food waste for different product groups	Chosen value
Cereals and potatoes	(Song et al., 2015) for HH CH: bread 1%, rice 2% (Xu, 2005) for CA BEI: rice 11–14%, noodles 5–13% (Liu, 2014) for CA LHA rice 18%, noodles 25% (Zhan, 1995) for CO CH: wheat 4%, rice 7%, corn 7% (Vanham et al., 2015) for CO EU cereals 17%, potatoes 26%	10%
Sugar	sugar HH CH 5% (Song et al., 2015); sugar CO EU 5–10% (Vanham et al., 2015)	5%
Crop oils	Vegetable oils HH CH 37% (Song et al., 2015); vegetable oils CO EU 5% (Vanham et al., 2015)	5%
Vegetables	vegetables HH CH 7% (Song et al., 2015); vegetables CA BEI 12–16% (Xu, 2005); vegetables CA LHA 17% (Liu, 2014); vegetables CO EU 26% (Vanham et al., 2015)	10%
Fruit	fruit HH CH 6% (Song et al., 2015); fruit CA BEI 7–9% (Xu, 2005); fruit CA LHA 6% (Liu, 2014); fruit CO EU 26% (Vanham et al., 2015)	6%
Pulses, nuts and oilcrops	legumes HH CH 2% (Song et al., 2015); pulses CO EU 5% (Vanham et al., 2015)	5%
Meat	(Song et al., 2015) for HH CH: beef 1%, pork 2%, poultry 7% (Xu, 2005) for CA BEI: pork 10–12%; beef 7–9%, poultry 8–11% (Liu, 2014) for CA LHA: pork 15%; beef 17%, poultry 14% (Vanham et al., 2015) for CO EU: meat 15%	7%
Offals edible		7%
Fish and seafood	aquatic products HH CH 8% (Song et al., 2015); aquatic products CA BEI 8–12% (Xu, 2005); aquatic products 18% CA LHA (Liu, 2014); fish CO EU 15% (Vanham et al., 2015)	8%
Animal fats	Animal fats CO EU 5% (Vanham et al., 2015)	5%
Milk and milk products	milk HH CH 0.3% (Song et al., 2015); milk CO EU 7% (Vanham et al., 2015)	5%
Eggs	eggs HH CH 2% (Song et al., 2015); eggs CA BEI 7–13% (Xu, 2005); eggs CA LHA 21% (Liu, 2014); pulses CO EU 5% (Vanham et al., 2015)	5%
Stimulants	stimulants CO EU 5–10% (Vanham et al., 2015)	5%
Spices	spices CO EU 5–10% (Vanham et al., 2015)	5%
Alcoholic Beverages	Liquor CA LHA 10% (Liu, 2014); alcoholic beverages CO EU 5% (Vanham et al., 2015)	5%

To determine the direct water use of Hong Kong citizens (i.e. the municipal WF), data on water use were assembled from the Water Supplies Department of Hong Kong. More particularly, data on total and per capita municipal and domestic water abstraction were obtained from its annual reports (Hong Kong Water Supplies Department, 2015). Municipal water use (326 l/cap/d abstraction in 2013) includes domestic water use (192 l/cap/d in 2013) and commercial water use (or water for services). Commercial water use includes the water use of small businesses, hotels, offices, hospitals, schools and other institutions. Municipal water use also represents water for non-permanent residents (like commuters or tourists). In municipal water use we also include water use for flushing of pipes as well as water losses.

It is important to distinguish between water abstraction (or water withdrawal) and water consumption (or consumptive water use). The difference between the two is returned water. The direct water use or blue WF (blue water consumption) of municipal water use is calculated based upon the municipal abstraction in 2013 (326 l/cap/d). The major sources of actual consumption consist of water lost through evapotranspiration from leaking supply and sewerage pipes, from watering plants and recreational areas, washing streets, and garden plots. The extent of the evapotranspiration also depends on climatic conditions. For Hong Kong, because generally all wastewater is directly discharged in the sea, all abstraction can be regarded as “consumptive use”.

### 2.2 Diets

Apart from the reference situation (1996–2005), we analyse 3 diet scenarios:

• The healthy diet (scenario HEALTHY): we use the food-based-dietary guidelines of the Chinese Nutrition Society (2015), as displayed in the Chinese food guide pagoda (Fig. 1). The third and last version of the Chinese FBDG was compelled by the Chinese Nutrition Society in 2007, and proclaimed by the Ministry of Health in early 2008 (Ge, 2011). The second version dates from 1997. In Fig. 1 we show the latest version but also the values from 1997. The revised pagoda kept the previous food grouping and placement but altered the amount of some food groups. Especially for milk (products) the new recommendation is much higher. Based upon specific energy requirements for different population groups (Ge, 2011, Ge et al., 2007) and the population distribution of Hong Kong (Hong Kong Statistics, 2015a), we calculate an average recommended energy requirement of 2122 kcal/cap/d (2204 kcal/cap/d for men and 2049 kcal/cap/d for women). Recommended food product intake values for this energy requirement are listed in Table 4.

**Table 4:** Chosen food product intake values for HEALTHY, based upon Chinese FBDG (Chinese Nutrition Society, 2015) and a – based upon population statistics (Hong Kong Statistics, 2015a) – computed average energy intake of 2122 kcal/cap/d. Source for sugar: (WHO, 2003). Source for alcohol: (Hong Kong Department of Health, 2013).

	HEALTHY recommended amounts	
	g/cap/d	kg/cap/yr
Cereals, rice, potatoes	325	118.6
Sugar	Max 60	Max 21.9
Vegetables	375	136.9
Fruit	300	109.5
Pulses, nuts and oilcrops	40	14.6
Meat	75	27.4
Offals	Included in meat	
Fish and seafood	Fish and shrimp 75	27.4
Animal fats and crop oils	25	9.1
Milk and milk products	300	109.5
Eggs	40	14.6
Stimulants	No specific recommendations	
Spices	No specific recommendations	
Alcoholic Beverages	Max 20 pure alcohol for men (2 standard drinks) and max 10 pure alcohol for women (1 standard drink)	Max 7.3 pure alcohol for men (2 standard drinks) and max 3.7 pure alcohol for women (1 standard drink)
• The pesco-vegetarian diet (scenario PESCO-VEG): identical as HEALTHY, but all meat and offals are substituted with products from the product group pulses. Animal fats are substituted with crop oils. All these substitutions results in the same total kcal and protein values.
• The vegetarian diet (scenario VEG): identical as PESCO-VEG, but all fish is substituted with products from the product group pulses (with the same kcal and protein values)

### 2.3 Impact assessment

We make a general evaluation/identification of blue water scarcity related to the production of food items in countries where Hong Kong imports from. Thereby we can identify the level of water related risk to Hong Kong’s food security.

To achieve this, we use the blue water scarcity database described in Mekonnen and Hoekstra (2016), who assessed global blue water scarcity (or water stress) on a monthly basis at the level of grid cells of 30 × 30 arc minutes. Blue water scarcity (WS) is here defined as the ratio between consumptive blue water use (the blue WF) and environmentally available water, the latter being available blue water resources minus environmental flow requirements (EFRs). This definition of water stress is in line with the requirements of a water stress indicator (Vanham et al., 2018). A widely used definition of environmental flow is “the quality, quantity, and timing of water flows required to maintain the components, functions, processes, and resilience of aquatic ecosystems which provide goods and services to people” (Hirji, 2009). Regarding EFRs, Mekonnen and Hoekstra (2016) adopted the presumptive environmental flow standard, according to which 80% of the natural runoff is allocated as EFR; the remaining 20% can be considered as blue water available for human use without affecting the integrity of downstream water-dependent ecosystems and livelihoods. To account for temporal changes in water availability and demand, Mekonnen and Hoekstra (2016) provide their analysis on a monthly level. Annual average monthly blue WS per grid cell was estimated by averaging the monthly scarcity values. Blue WS is called “low” when in a grid cell WS < 1 (when the blue water footprint does not exceed blue water availability, in which case EFRs are met), “moderate” when 1 ≤ WS ≤ 1.5, significant when 1.5 < WS ≤ 2 and “severe” when WS > 2.

In our study, we identify whether food items Hong Kong imports, have a blue WF located in water scarce regions. We then evaluate the average national WS in grid cells where a particular food item is produced, for the main import source countries of Hong Kong. We identify grid cells where these food items are produced by means of the data of:

• The GIS-database on the WF_prod,bl_ of main crop products from Mekonnen and Hoekstra (2011). This database includes maize, wheat, rice, sugarcane and soybeans. Only grid cells with a WF_prod,bl_ are selected. Grid cells with only a WF_prod,gn_ component are not selected to compute average national WS for a product item in countries Hong Kong imports from.
• The GIS-database on crop production from Monfreda et al. (2008). This database includes additional products like oranges, grapes or specific tree nuts (pistachios or almonds). Here we select all grid cells in a country, because we lack information on which grid cell is irrigated or not.

## 3 Results and discussion

### 3.1 The reference situation

**Fig. 2:**
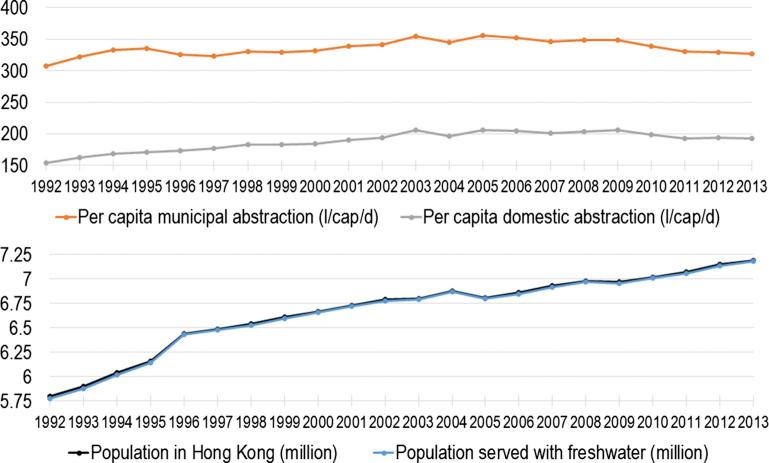
Direct water use Hong Kong: (Top) Per capita municipal and domestic water abstraction/withdrawal in l/cap/d; (Bottom) Total population and population served with fresh water (in million). Data source: (Hong Kong Water Supplies Department, 2015).

As shown in Fig. 2, both the municipal and domestic water withdrawals of Hong Kong have – after a decade of steady increase – stabilized and then gradually decreased since 2003, although the population has not stopped to grow. Hong Kong has indeed put a lot of effort in decreasing its direct water use. Therefore the municipal WF (blue water) is calculated based upon the municipal water abstraction of 2013 (326 l/cap/d) and is 326 l/cap/d. According to the database of Mekonnen and Hoekstra (2016), annual water stress in the Hong Kong area, where the city takes it direct water use from, is low (<1). Particular monthly values are however higher. This database is course in its resolution, and local WS values can differ.

**Fig. 3:**
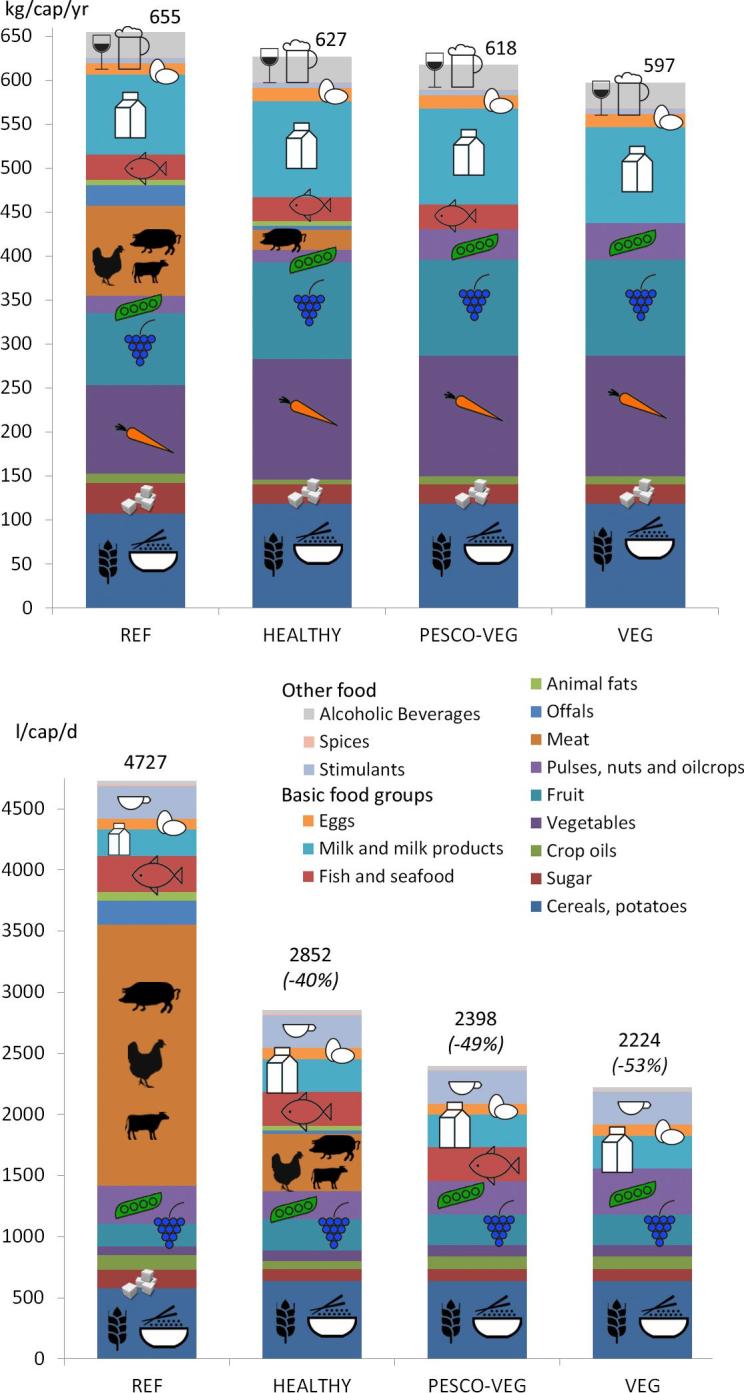
(Above) Food intake in Hong Kong (in kg/cap/yr) for REF and the three scenarios; (Bottom) The green + blue WF of consumption for edible agricultural products (WF_cons, gn+bl_ of food) (in l/cap/d) of Hong Kong for REF and the three scenarios. (For interpretation of the references to colour in this figure legend, the reader is referred to the web version of this article.)

The WF_cons, gn+bl_ related to food consumption in Hong Kong amounts to 4727 l/cap/d (Fig. 3). This indirect water use is about 15 times the amount of the direct water use of Hong Kong. Especially the intake of meat contributes a large part to this indirect water use.

Interesting to see is the typical urban food consumption behaviour of Hong Kong as compared to the food consumption behaviour in China as a whole (Fig. S1 and Table S1). Total food supply to China is 657 kg/cap/yr and to Hong Kong 794 kg/cap/yr, with a large difference in the relative composition of the different food groups. In China supply of cereals (including potatoes) (233 kg/cap/yr) and vegetables (230 kg/cap/yr) is much higher than in Hong Kong (131 respectively 112 kg/cap/yr). But in Hong Kong the food supply of sugar, crop oils, fruit, meat and offals, fish and seafood and milk and milk products are much higher. Especially the amounts of meat (154 kg/cap/yr), offals (24 kg/cap/yr) and fish and seafood (63 kg/cap/yr) are extremely high, even as compared to the EU average (85 respectively 4 and 21 kg/cap/yr). As a matter of fact, meat supply in Hong Kong is amongst the highest in the world.

As a result, the WF_cons, gn+bl_ (4727 l/cap/d) related to food consumption in Hong Kong is almost double the Chinese WF_cons, gn+bl_ (2413 l/cap/d). That is because it requires a lot of water to produce product groups like meat, fish, sugar or milk (i.e. their WF_prod_ is very high as displayed in Table S2).

### 3.2 Diet scenarios

A comparison of the current Hong Kong diet with the scenario HEALTHY shows that the current average diet is not healthy. As displayed in Table 5, the intake of some product groups should be increased (vegetables, fruits, but also milk and milk products) and of others decreased (sugar, crop oils, meat, offals and animal fats). The intake of fish and seafood is as recommended. Current energy intake (2960 kcal/cap/d), protein intake (114.1 g/cap/d) and fat intake (127.5 g/cap/d) related to the basic food groups (not taking stimulants, alcoholic beverages or spices into account) will decrease with a healthy diet to 2260 kcal/cap/d, 72.4 g/cap/d and 66.4 g/cap/d respectively. This energy intake reaches the target energy intake of 2122 kcal/cap/d quite well. The PESCO-VEG and VEG scenarios show about the same energy, protein and fat values as the HEALTHY scenario.

**Table 5:** Reference and scenario food intake values per product group in terms of weight (kg/yr), energy (kcal/d), protein (g/d) and fat (g/d). All values per capita. Total 1 = basic food groups; total 2 = total 1 + other food.

Product group	Weight (kg/yr)				Energy (kcal/d)				Protein (g/d)				Fats (g/d)			
	REF	HEALTHY	PESCO-VEG	VEG	REF	HEALTHY	PESCO-VEG	VEG	REF	HEALTHY	PESCO-VEG	VEG	REF	HEALTHY	PESCO-VEG	VEG
Cereals, potatoes	107.6	118.6	118.6	118.6	865	954	954	954	16.9	18.6	18.6	18.6	2.1	2.3	2.3	2.1
Sugar	34.9	21.9	21.9	21.9	301	189	189	189	0.0	0.0	0.0	0.0	0.0	0.0	0.0	0.0
Crop oils	10.5	5.7	9.1	9.1	252	137	219	219	0.0	0.0	0.0	0.0	28.5	15.5	24.8	24.8
Vegetables	100.7	136.9	136.9	136.9	108	146	146	146	4.8	6.5	6.5	6.5	0.9	1.2	1.2	1.2
Fruit	81.9	109.5	109.5	109.5	84	112	112	112	1.1	1.4	1.4	1.4	0.5	0.6	0.6	0.6
Pulses, nuts, oilcrops	19.3	14.6	35.1	41.4	171	129	301	383	7.2	5.4	17.2	29.0	12.9	9.7	11.2	18.1
Meat	102.7	22.4	0.0	0.0	697	152	0	0	46.2	10.1	0.0	0.0	55.2	12.1	0.0	0.0
Offals, edible	22.8	5.0	0.0	0.0	69	15	0	0	11.3	2.5	0.0	0.0	2.0	0.4	0.0	0.0
Animal fats	6.3	5.0	0.0	0.0	110	87	0	0	0.1	0.1	0.0	0.0	12.3	9.7	0.0	0.0
Fish and seafood	28.9	27.4	27.4	0.0	87	83	83	0	14.4	13.6	13.6	0.0	2.5	2.3	2.3	0.0
Milk and milk products	90.5	109.5	109.5	109.5	164	198	198	198	8.0	9.6	9.6	9.6	6.9	8.3	8.3	8.3
Eggs	13.3	14.6	14.6	14.6	53	58	58	58	4.2	4.6	4.6	4.6	3.8	4.1	4.1	4.1
**Total 1**	**619.3**	**591.1**	**582.6**	**561.5**	**2960**	**2260**	**2260**	**2260**	**114.1**	**72.4**	**71.6**	**69.8**	**127.5**	**66.4**	**55.0**	**59.3**
Stimulants	6.0	6.0	6.0	6.0	32	32	32	32	0.9	0.9	0.9	0.9	2.4	2.4	2.4	2.4
Alcoholic beverages	28.5	28.5	28.5	28.5	49	49	49	49	0.3	0.3	0.3	0.3	0.0	0.0	0.0	0.0
Spices	0.9	0.9	0.9	0.9	9	9	9	9	0.3	0.3	0.3	0.3	0.3	0.3	0.3	0.3
**Total 2**	**654.8**	**626.5**	**618.0**	**596.9**	**3050**	**2350**	**2350**	**2350**	**115.6**	**73.9**	**73.1**	**71.3**	**130.2**	**69.1**	**57.7**	**62.1**

A shift to a healthy diet would reduce the WF_cons, gn+bl_ substantially by 40%, from 4727 l/cap/d to 2852 l/cap/d (Fig. 3). Especially the decrease in meat intake has a major effect on the WF_cons, gn+bl_ reduction, as the meat related WF_cons, gn+bl_ decreases from 2134 l/cap/d to 466 l/cap/d. The PESCO-VEG scenario reduces the WF_cons, gn+bl_ by 49% to 2398 l/cap/d. The VEG scenario decreases the WF_cons, gn+bl_ by 53% to 2224 l/cap/d.

**Fig. 4:**
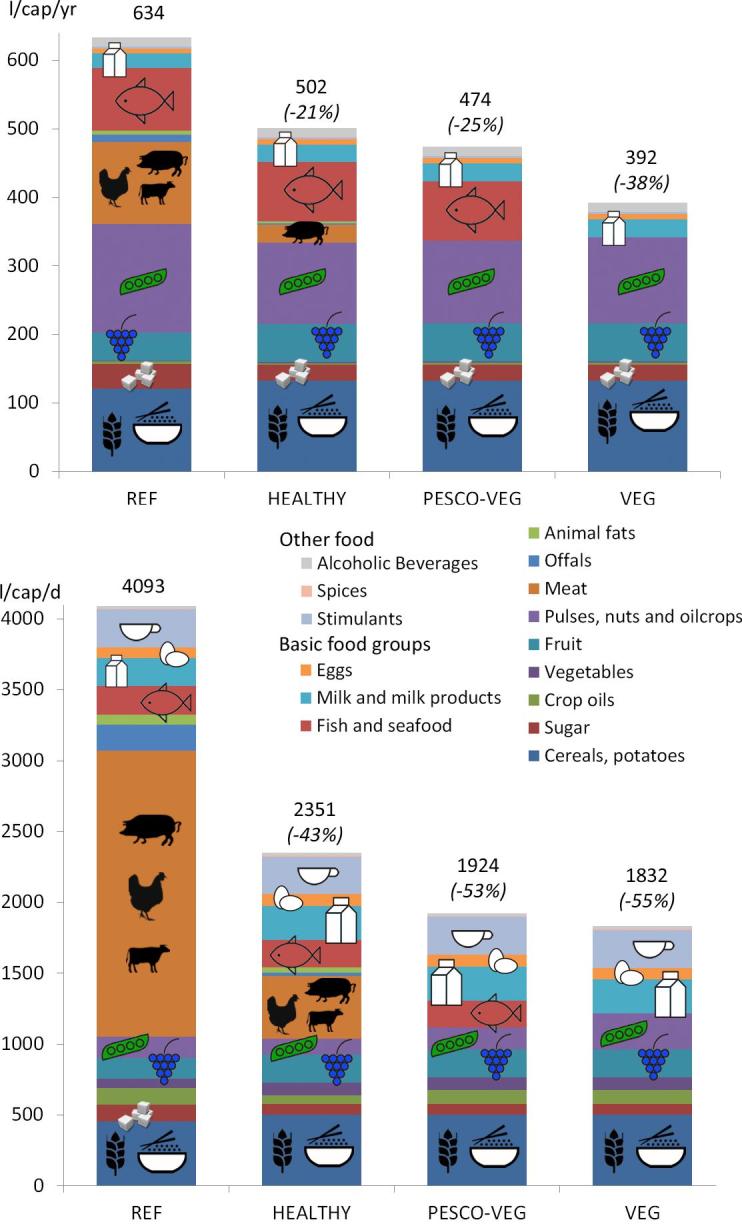
(Above) The blue WF of consumption for edible agricultural products (WF_cons, bl_ of food) (in l/cap/d) of Hong Kong for REF and the three scenarios; (Bottom) The green WF of consumption for edible agricultural products (WF_cons, gn_ of food) (in l/cap/d) of Hong Kong for REF and the three scenarios. (For interpretation of the references to colour in this figure legend, the reader is referred to the web version of this article.)

When the blue and green WF components are assessed separately, the WF_cons_ also decreases for all scenarios with respect to the current situation (Fig. 4). For the WF_cons, gn_, the observations are generally the same as for the WF_cons, gn+bl_. However, for the WF_cons, bl_, the dominance of the meat fraction disappears. Decreases in the WF_cons, bl_ of meat and offals, pulses, nuts and oilcrops as well as sugar for all three diet scenarios contribute mainly to the overall decrease in the total WF_cons, bl_. Increases in the WF_cons, bl_ of fruit and cereals partly compensate for these decreases, in all three diet scenarios.

Recommendations on the intake of milk and milk products by the Chinese Nutrition Society increased from 100 g/cap/d in 1997 to 300 g/cap/d in 2007 (Fig. 1). We used the most recent values. These lead to high WF_cons_ amounts (Fig. 3, Fig. 4 HEALTHY), as it requires a lot of water to produce milk (Table 2). The protein and fat intake with this milk intake is also high (Table 5). An increase to 500 g/cap/d would increase the WF_cons_ of healthy significantly. When visiting a dairy farm in Chongqing in April 2006, Premier Wen Jiabao made the “500 Gram Dairy Declaration”, stating “I have a dream that every Chinese, especially children, could have 0.5 kg of dairy products every day” (Jiabao, 2008).

### 3.3 Impact of Hong Kong’s food consumption WF

The total WF_cons,bl_ related to food consumption in Hong Kong is 634 l/cap/d, which equals about 1.6 km^3^/yr of blue water (Table 6). Some main food items are responsible for the highest fractions of this amount, as also displayed in Fig. 4.

**Table 6:** Food items in the Hong Kong diet with high WF_cons, bl_ amounts. Reference population of 6737 * 10^3^ people (average of 1996–2005).

Food item	WF_cons, bl_		Main countries of import	Annual average of monthly blue water scarcity (WS) for grid cells where food item is produced (with irrigation), national average
	l/cap/d	km^3^/yr	(% of total volume)	
Wheat	57	0.140	64% China	China 2.4, according to WF_prod, bl_ database for wheat of (Mekonnen and Hoekstra, 2011)
Rice (milled eq.)	62	0.152	55% Thailand, 30% Vietnam, 9.2% China, 1.4% Australia, 1.1% USA	Thailand 3.2, Vietnam 2.0, China 1.5, Australia 4.8, USA 1.4, according to WF_prod, bl_ database for rice of (Mekonnen and Hoekstra, 2011)
Sugar (Raw Equivalent)	36	0.088	All sugar from sugarcane assumed. Sugar from Rep. of Korea originates from Australia	Australia 3.6, according to WF_prod, bl_ database for sugarcane of (Mekonnen and Hoekstra, 2011)
Tree nuts	157	0.385	20% USA pistachios, 17% USA almonds and 28% USA other nuts, 18% Rep. of Iran pistachios, 18% worldwide nut mix	USA almonds 3.5, USA pistachios 4.2, Rep. of Iran pistachios 3.0, according to the crop production database for almonds and pistachios of (Monfreda et al., 2008)
Oranges, Mandarines	9	0.021	20% South Africa, 54% USA, 11% Australia	South Africa 3.8, USA 4.0, Australia 5.4, according to the crop production database for oranges of (Monfreda et al., 2008)
Grapes and products (excl wine)	14	0.035	39% Chile, 26% USA	Australia 4.9, Chile 1.2, USA 1.4, France 0.3, according to the crop production database grapes of (Monfreda et al., 2008)
Wine	13	0.032	32% France, 15% Australia, 13% USA	
Bovine Meat	19	0.048	38% Brazil, 28% USA	Maize and soybeans as feed crops are taken as proxy for the WF_prod,bl_ of these livestock products in countries of orgin. It is assumed that feed crops are produced in these countries themselves. *Maize*: Brazil neglectable amount of irrigated maize, USA 2.4, China 3.0, Spain 3.1, Germany 0.3, according to WF_prod, bl_ database for maize of (Mekonnen and Hoekstra, 2011) Soybeans: Brazil 0.2, USA 1.8 China 2.5, Spain 3.8, Germany neglectable amount of irrigated soybeans, according to WF_prod, bl_ database for maize of (Mekonnen and Hoekstra, 2011)
Pigmeat	64	0.158	24% China, 18% Brazil, 9% Spain, 8% USA, 8% Germany	
Poultry Meat	32	0.079	28% Brazil, 32% USA	
Offals	9	0.022	Offals cattle (35% of all offals) 65% Brazil, Offals pigs (63% of all offals) 25% Germany, 17% USA	
Eggs	7	0.018	59% China, 23% USA	
Milk	21	0.052	23% Netherlands, 15% China, 13% Australia	
Freshwater Fish	71	0.175	No data	High value due to pond evaporation and feed input
Demersal, pelagic and other marine fish	16	0.038	No data	Only feed input
Remaining food items	47	0.115		
All products	634	1.558		

**Fig. 5:**
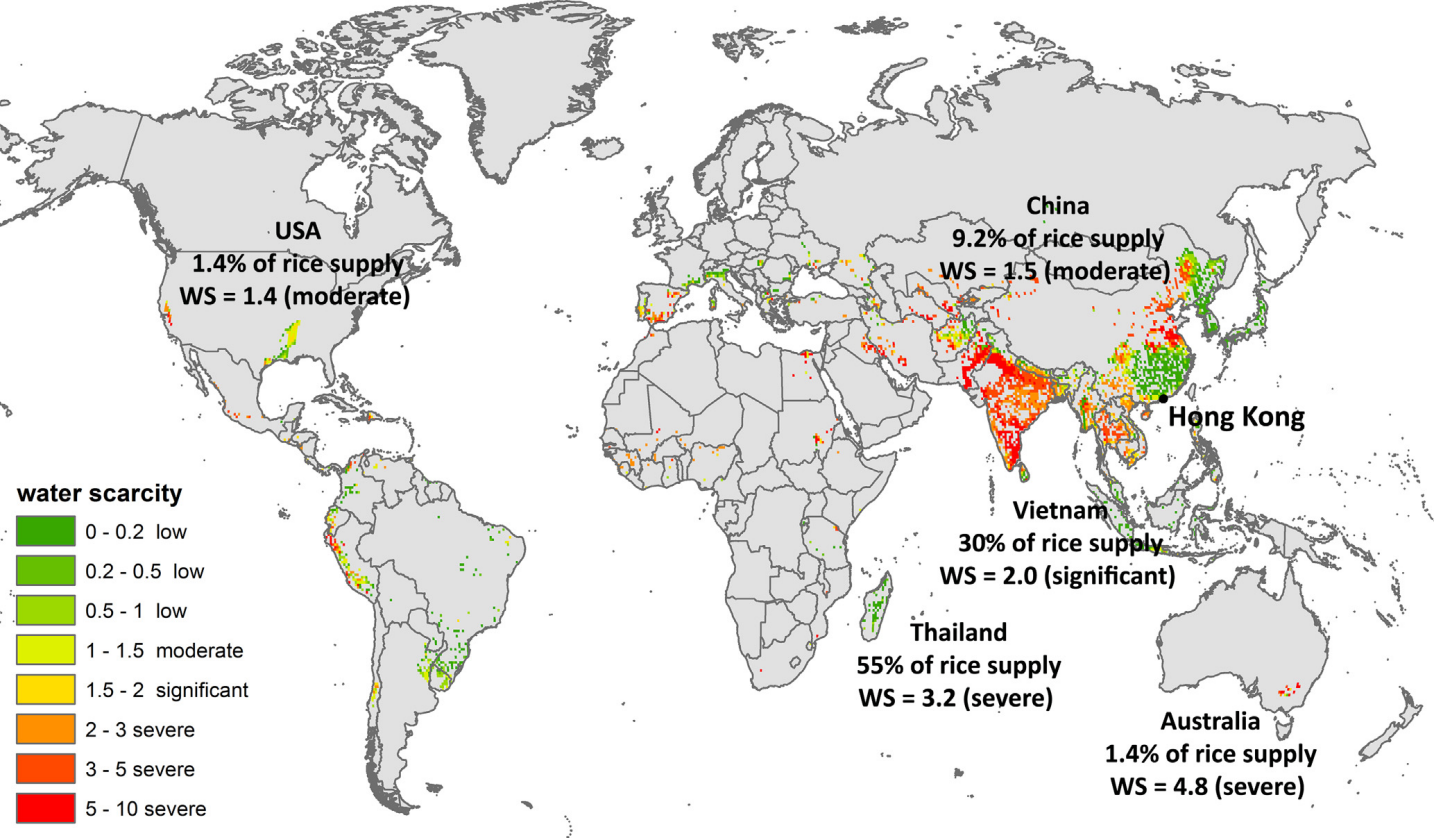
Annual average of monthly blue water scarcity (WS), according to (Mekonnen and Hoekstra, 2016), in grid cells where rice has a blue WF, according to data from (Mekonnen and Hoekstra, 2011). (For interpretation of the references to colour in this figure legend, the reader is referred to the web version of this article.)

Rice, with a WF_cons,bl_ of 62 l/cap/d or 0.152 km^3^/yr has a relatively high value. We chose this crop as more detailed example of Hong Kong’s food consumption WS impact, as it is a Chinese staple crop with a relatively high WF. The rice imported by Hong Kong is partly produced under conditions of water scarcity (WS) (Table 6 and Fig. 5). Thai rice, which represents 55% of consumed rice in Hong Kong, is e.g. produced under an average WS condition of 3.2 (severe WS). Vietnamese rice (30% of consumed rice in Hong Kong), is produced under a WS of 2.0 (significant). For Chinese, Australian and US rice (9.2%, 1.4% and 1.1% of consumed rice), WS values are 1.5 (moderate), 4.8 (severe) and 1.4 (moderate). Due to Hong Kong’s relatively low total rice supply amount of 343 ∗ 10^3^ tonnes/yr on the global market, these imports represent generally only a low fraction of the total WF_prod,bl_ of rice in the countries of origin. Thai rice consumed in Hong Kong has a WF_cons,bl_ of 0.102 km^3^/yr, which only represents about 1% of the total Thai WF_prod,bl_ of rice (9.682 km^3^/yr). Nevertheless, this shows the unsustainable production of rice in the countries Hong Kong imports rice from. Especially in Australia, with a WS value of 4.8 (severe) in the rice producing region of the Murray–Darling Basin (Khan et al., 2009), it is questionable if this practice can be maintained in the long run. From the viewpoint of Hong Kong, rice import might be at risk from this location.

**Fig. 6:**
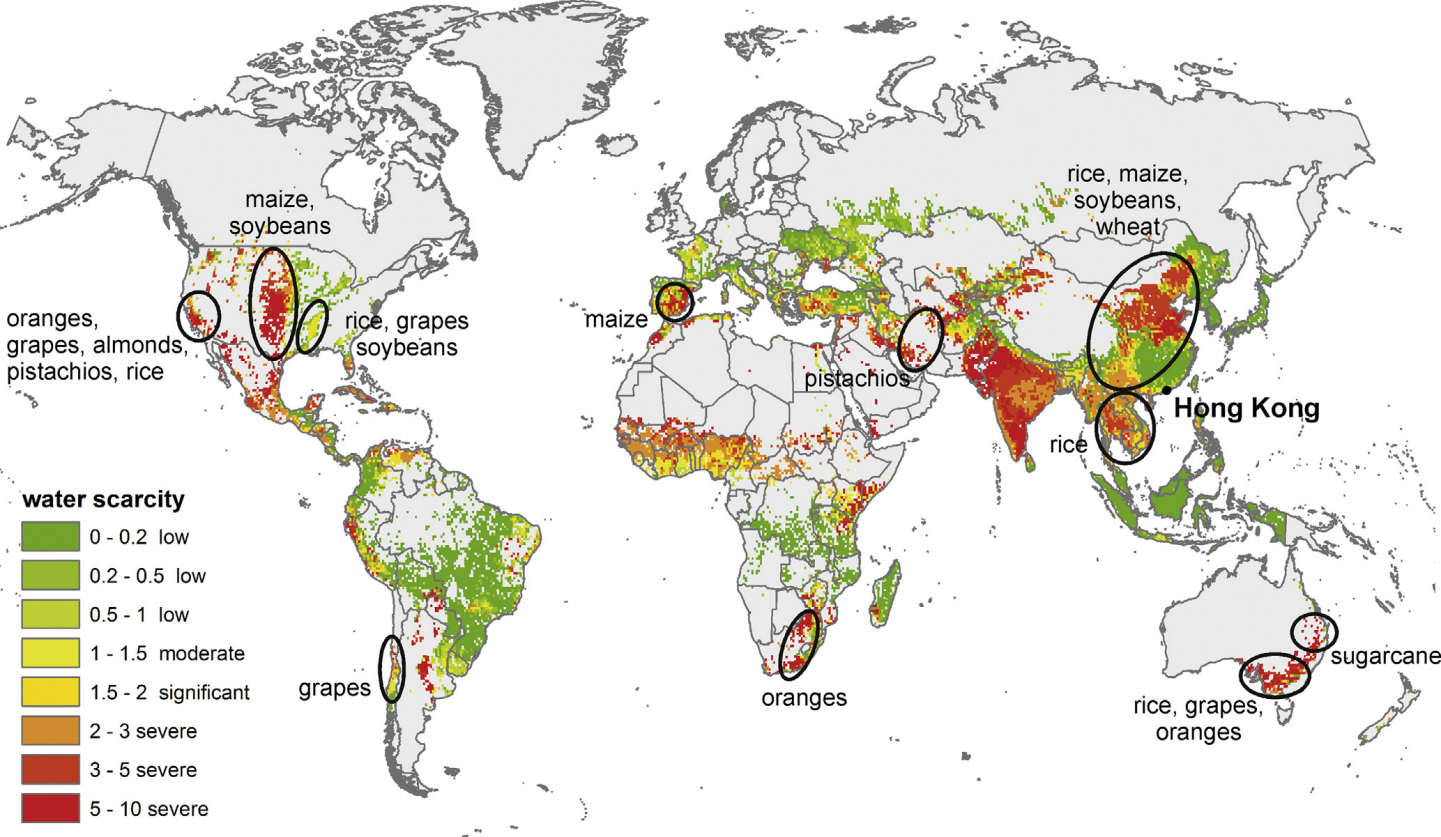
Identification of WS hot spots: High values in the annual average of monthly blue water scarcity (WS) (Mekonnen and Hoekstra, 2016), for food items with a high WF_cons,bl_ Hong Kong imports. These food items are wheat, rice, sugarcane, specific tree nuts (pistachios and almonds), oranges, grapes, and maize and soybeans as proxy for livestock products. (For interpretation of the references to colour in this figure legend, the reader is referred to the web version of this article.)

A global map on WS related to the production of food products with a high WF_cons,bl_ consumed in Hong Kong is shown in Fig. 6. This includes wheat, rice, sugar, selected tree nuts (pistachios and almonds), oranges, grapes and maize and soybeans as proxy for livestock products (Table 6). The areas identified in the map are hotspots with moderate to severe water scarcity. It is clear that these imported products represent to a certain level a future water-related risk for food security in Hong Kong.

A reduction in this risk is achieved in the three diet scenarios, which all result in a decrease of the total WF_cons,bl_ (Fig. 4). Less meat intake results in a strong reduction of the total WF_cons,bl_. Also less intake of sugar and products from the group pulses, nuts and oilcrops contribute to a lower total WF_cons,bl_. Especially nuts like pistachios and almonds have very high WF_prod,bl_ values. On the other hand, the recommended increased intake of fruit compensates for this decrease. This is because in our analysis we kept the relative fractions of food items within their product groups constant for the diet scenarios. Choosing for an increased intake of fruit that has not such high WF_prod,bl_ values, is a smart way to tackle this issue.

### 3.4 Limitations and further research

The consumption of other goods leads to additional indirect water uses by Hong Kong citizens. In our study we only focus on the indirect water use related to food consumption. As such, the total Hong Kong WF_cons_ will be even higher when additional water uses will be included. This includes green and blue water resources for non-edible agricultural products (e.g. cotton for clothes), industrial goods or forestry (e.g. paper or wood for energy). A full list of water uses that lead to additional WF_cons_ components is given in Vanham (2016).

We did not separate between surface and groundwater use in our analysis, as recommended by Vanham et al. (2018). The WS data we used e.g. do not identify groundwater depletion, although the rapidly depletion of aquifers is a major global issue (Dalin et al., 2017, Scanlon et al., 2012, Wada et al., 2010), which poses a serious threat for global food security.

To assess the environmental sustainability of a WF, not only the impact (blue WS) needs to be addressed (Vanham et al., 2018), but also the water efficiency/productivity of a WF. Sustainable development goal 6.4 states: “*By 2030, substantially increase water-use efficiency across all sectors and ensure sustainable withdrawals and supply of freshwater to address water scarcity and substantially reduce the number of people suffering from water scarcity*”. A way to assess water productivity of agricultural products is to work with WF benchmarks (which include green and blue water), as e.g. described in Mekonnen and Hoekstra (2014). Recently, a study addressing these 2 issues for the WF of the UK has been published (Hoekstra and Mekonnen, 2016). In addition, by not integrating the grey WF component, water quality has not been taken into account in our study. Increasing water productivity often comes at the expense of decreasing water quality in agriculture, an important trade-off. These issues are subject to further research for the WF_cons_ of Hong Kong.

There is a need for better food waste statistics for the city of Hong Kong. Our selection of food waste fractions for product groups (corr2 values in Table 2) is based upon literature values which have not a Hong Kong focus. These were however best available data. Also Liu et al. (2013) indicate that there is a lack of detailed food waste data in China. We only found one statistic on consumer food waste for Hong Kong, i.e. the Hong Kong Environmental Protection Department (2015) states that in 2014 a total of 2608 tonnes per day of domestic food waste (from households) were disposed of in landfills. This equals about 132 kg/cap/yr. We calculated a consumer (household and catering sector) food waste of 83 kg/cap/yr. This value results from the difference between the total food intake (655 kg/cap/yr) and the food consumption (retail product) (706 kg/cap/yr) plus the waste resulting from the cleaning of fish (31 kg/cap/yr), values displayed in Table 2. We can thus conclude that our corr2 values are conservative (underestimating). A food waste quantification campaign in Hong Kong (with differentiation between households and the catering sector as well as between different product groups) would therefore be very valuable. With such data this study could be even more refined.

## 4 Conclusions

During the last decade, direct urban water use has decreased in many modern cities. In Hong Kong, municipal water abstraction (the municipal or direct water footprint) has decreased from 355 l/cap/d in 2005 to 326 l/cap/d in 2013, although its population steadily increased during this period. Citizens and policy makers at the urban level are generally well informed on their direct water use and the possibilities for more sustainable water use.

This direct water use represents however only about 1/15th of the WF related to food consumption (WF_cons, gn+bl_) in Hong Kong (4727 l/cap/d) (Fig. 3). Meat consumption accounts for 45% (2134 l/cap/d) of this total value. Most of this amount represents green water (4093 l/cap/d) (Fig. 4). Blue water accounts for 634 l/cap/d (about 1.6 km^3^/yr).

This blue water amount is partly imported through food products produced within a water scarcity setting (WS). Most rice consumed in Hong Kong e.g., is produced under moderate to severe WS in Thailand, Vietnam, China, the USA or Australia (Fig. 5). Other food items consumed in Hong Kong with a high WF_cons,bl_ contributing to local WS are wheat, sugar, selected tree nuts (pistachios and almonds), oranges, grapes and livestock products (Table 6 and Fig. 6). To some extent, they pose a water-related risk to food security in Hong Kong. It can happen in future, that producing countries will not export these product anymore, because of several reasons (e.g. focus on domestic food security, addressing local water scarcity, depleting aquifers, less water availability due to climate change, decreased production quantities …).

The current average diet in Hong Kong is not healthy, according to Chinese dietary guidelines. It contains too much meat, offals, sugar, crop oils and animal fats and not enough vegetables, fruit and milk (products). The intake of meat (including offals) needs to be reduced by 79%. Overall energy (2960 kcal/cap/d for basic food groups) and protein (114.1 g/cap/d) intakes are too high and need to be reduced. We show that all three diet scenarios come close to the recommended energy requirement of 2122 kcal/cap/d, which is calculated based upon the population distribution of Hong Kong. They also achieve more recommendable protein and fat intake levels.

The diet scenarios result in substantial WF reductions. The WF_cons, gn+bl_ reduces 40% for HEALTHY, 49% for PESCO-VEG and 53% for VEG (Fig. 3). The WF_cons, gn_ reduces 43% for HEALTHY, 53% for PESCO-VEG and 55% for VEG (Fig. 4). For the blue WF the reductions are lower. The WF_cons, bl_ reduces 21% for HEALTHY, 25% for PESCO-VEG and 38% for VEG. These WF_cons, bl_ reductions contribute to a decrease in the water-related risk for food security in Hong Kong.

These diet scenarios present a win–win situation, because 1) they result in a healthier lifestyle for Hong Kong citizens (where 47% of the population is already overweight or obese), 2) it is expected they save in healthcare costs and 3) they also save large quantities of the precious resource water, resulting in a decreased water-related risk for Hong Kong food security.
