# Supplementary material for: Cities as hotspots of indirect water consumption: The case study of Hong Kong
Source: J Hydrol (Amst). 2019 Jun;573:1075–86. doi: 10.1016/j.jhydrol.2017.12.004 (PMC6588220; doi:10.1016/j.jhydrol.2017.12.004)
Supplement: Supplementary data 1 [file mmc1.pdf]

SUPPLEMENTARY MATERIAL

Table S1: Reference food supply to consumers amounts per product group (in kg/cap/yr) of the EU, China and Hong Kong

	Food supply (kg/cap/yr)		
	China	Hong-Kong	EU
Cereals, potatoes	233	131	206
Sugar	7	37	40
Crop oils	7	11	18
Vegetables	230	112	121
Fruit	45	87	99
Pulses, nuts and oilcrops	9	20	10
Meat	44	154	85
Offals	3	24	4
Fish and seafood	24	7	21
Animal fats	2	63	13
Milk and milk products	13	95	234
Eggs	15	14	12
Stimulants	0.4	6	7.5
Spices	0.3	1	0.5
Alcoholic Beverages	25	30	109
**SUM**	**657**	**794**	**981**

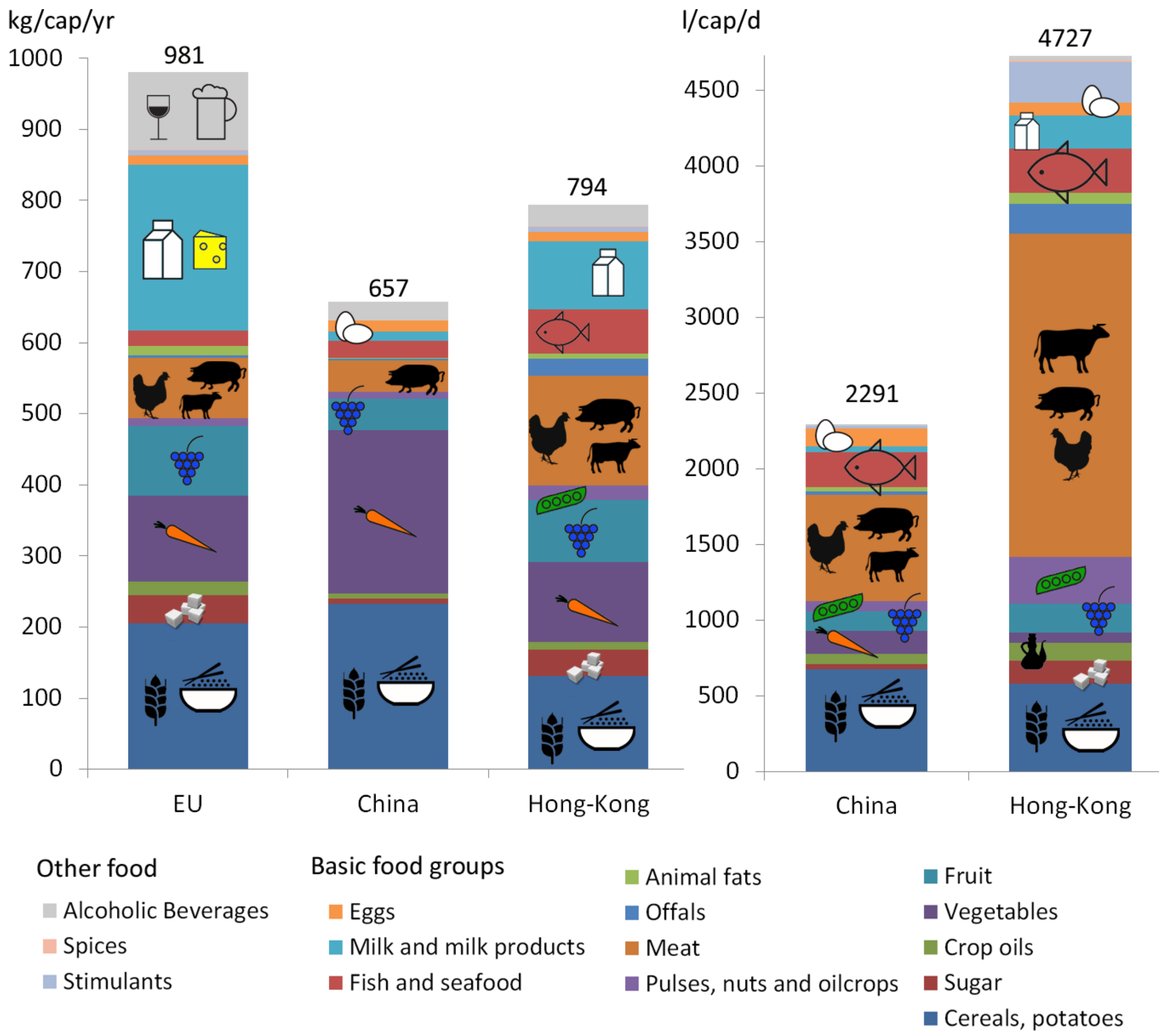

Figure S1: (Left) The reference food supply to consumers (in kg/cap/yr) of the EU, China and Hong Kong; (Right) The reference green+blue WF of consumption for edible agricultural products ($WF_{cons, gn+bl}$ of food) (in l/cap/d) for China and Hong Kong.

Table S2: Details for food items consumed in Hong Kong: Average annual FAO FBS values for 1996-2005 or 2010-2013, in 10^3 tonnes/year. Indication of main countries of import, based upon the FAO detailed trade matrix. Calculated green and blue WFprod and WFcons of food consumed in Hong Kong. PROD= production; IMP= import; EXP= export; PROC = processing; ROW= Rest Of World; HK= Hong Kong.

Food item	PROD (10^3 tonnes)	IMP (10^3 tonnes)	EXP (10^3 tonnes)	Domestic supply (10^3 tonnes)	FEED/PROC/OTHER (10^3 tonnes)	Food supply (10^3 tonnes)	Food supply (kg/cap/yr)	Main countries of import (2010-2013)	Calculated WF_{prod} of food consumed in Hong Kong (l/kg)			WF_{cons} in Hong Kong (l/cap/d)			Comments
									gn	bl	gn+bl	gn	bl	gn+bl	
**Cereals**															
Wheat and products	0	417	77	340	PROC 1	333	49.5	64% China, 36% ROW	985	421	1407	133	57	191	Food-related wheat consumption in HK from China = (333*0.64)=213*10^3 tonnes, which equals 0.1 km^3 blue water or 0.2% of the Chinese blue WF_{prod} for wheat
Rice (Milled Equivalent)	0	370	21	348	0	343	51.0	55% Thailand, 30% Vietnam; 15% ROW	2141	442	2582	299	62	361	Food-related rice consumption in HK a) from Thailand = (343*0.55)=189*10^3 tonnes, which equals 0.102 km^3 blue water or 1.06% of the Thai blue WF_{prod} for rice and b) from Vietnam (343*0.30)=103*10^3 tonnes, which equals 0.03 km^3 blue water or 0.5% of the Vietnamese blue WF_{prod} for rice
Barley and products	0	16	4	11	PROC 11	1	0.1	67% USA, 33% ROW	1021	174	1195	0	0	0	Barley is used for domestic beer production. This use (11*10^3 tonnes) relates to 0.002 km^3 blue water or 0.1% of the US blue WF_{prod} for barley
Maize and products	0	71	7	65	FEED 30	30	4.5	78% USA, 22% ROW	616	67	683	8	1	8	
Rye and products	0	4	0	4	0	4	0.5	100% ROW	1419	25	1444	2	0	2	
Oats	0	9	4	5	0	4	0.6	53% China, 47% ROW	1070	92	1162	2	0	2	
**Starchy roots**															
Cassava and products	0	229	229	8	0	6	0.9	97% Thailand, 3% ROW	438	0	438	1	0	1	

Food item	PROD (10^3 tonnes)	IMP (10^3 tonnes)	EXP (10^3 tonnes)	Domestic supply (10^3 tonnes)	FEED/PROC/OTHER (10^3 tonnes)	Food supply (10^3 tonnes)	Food supply (kg/cap/yr)	Main countries of import (2010-2013)	Calculated WF_{prod} of food consumed in Hong Kong (l/kg)			WF_{cons} in Hong Kong (l/cap/d)			Comments
									gn	bl	gn+bl	gn	bl	gn+bl	
Potatoes and products	0	292	69	224	FEED 36, OTHER 15	170	23.8	65% USA, 35% ROW	150	12	162	10	1	11	
Sugar & Sweeteners															
Sugar (Raw Equivalent)	0	336	93	241	/	233	35	48% Republic of Korea, 15% CHINA, 37% ROW	890	378	1268	84	36	120	All sugar from sugarcane assumed. Sugar from Republic of Korea originates from Australia.
Pulses															
Beans	0	9	1	9		9	1.2	55% CHINA, 45% ROW	267	29	296	1	0	1	
Peas	0	3	1	1		1	0.2	84% CHINA, 16% ROW	522	25	547	0	0	0	
Treenuts															
Treenuts	0	375	289	94	OTHER 3	92	13	64% USA, 18% Iran, 18% ROW	3338	4407	7745	119	157	275	Import 20% USA pistachios, 17% USA almonds and 28% USA other nuts, 18% Rep. of Iran pistachios, 18% worldwide nut mix
Oilcrops															
Soyabeans	0	44	23	20	FEED 2	18	3	53% CANADA, 34% CHINA, 13% ROW	1891	94	1985	14	1	14	
Vegetable Oils															
Soyabean Oil	0	315	91	223	OTHER 189	35	5	38% Brazil, 26% USA, 37% ROW	3807	97	3904	54	1	55	
Groundnut Oil	0	11	3	8		8	1	46% China, 54% ROW	5222	319	5541	16	1	17	
Rape and Mustard Oil	0	50	13	36	OTHER 30	6	1	93% Canada, 7% ROW	4910	30	4940	11	0	11	
Maize Germ Oil	1	7	1	6		6	1	58% China, 42% ROW	1769	159	1928	4	0	5	
Vegetables															

Food item	PROD (10^3 tonnes)	IMP (10^3 tonnes)	EXP (10^3 tonnes)	Domestic supply (10^3 tonnes)	FEED/PROC/OTHER (10^3 tonnes)	Food supply (10^3 tonnes)	Food supply (kg/cap/yr)	Main countries of import (2010-2013)	Calculated WF_{prod} of food consumed in Hong Kong (l/kg)			WF_{cons} in Hong Kong (l/cap/d)			Comments
									gn	bl	gn+bl	gn	bl	gn+bl	
Tomatoes and products	0	27	3	24		22	3.1	53% China, 24% Italy, 23% ROW	137	24	160	1	0	1	
Onions	0	15	2	13		11	1.7	30% China, 70% ROW	176	33	209	1	0	1	
Vegetables, Other	37	851	33	856	OTHER 19	764	107	99% China, 1% ROW	218	4	222	64	1	65	
Fruits															
Oranges, Mandarines	0	256	69	188	OTHER 5	159	24	20% South Africa, 54% USA, 11% Australia, 15% ROW	262	131	393	17	9	26	
Lemons, Limes and products	0	23	3	19		19	2.8	39% South Africa, 36% USA, 25% ROW	311	130	442	2	1	3	
Grapefruit and products	0	17	2	15		15	2.2	19% China, , 81% ROW	437	76	513	3	0	3	
Bananas	0	56	13	43	OTHER 1	36	5.4	84% Philippines, 16% ROW	938	16	954	14	0	14	
Apples and products	0	101	34	67	OTHER 2	56	8.3	30% China, 40% USA, 30% ROW	466	147	613	11	3	14	
Pineapples and products	0	14	1	12		12	1.7	63% Philippines, 37% ROW	169	3	172	1	0	1	
Dates	0	4	2	2		2	0.3	94% China, 6% ROW	529	75	604	0	0	0	
Grapes and products (excl wine)	0	116	63	53	OTHER 2	40	6.0	39% Chile, 26% USA, 35% ROW	565	878	1443	9	14	24	
Fruits, Other	4	471	191	284	OTHER 9	247	37	73% Thailand, 27% ROW	894	131	1025	90	13	103	
Stimulants															
Coffee and products	0	31	5	26		24	3.4	21% Malaysia, 18% China, 13%	16287	57	16344	152	1	153	

Food item	PROD (10^3 tonnes)	IMP (10^3 tonnes)	EXP (10^3 tonnes)	Domestic supply (10^3 tonnes)	FEED/PROC/OTHER (10^3 tonnes)	Food supply (10^3 tonnes)	Food supply (kg/cap/yr)	Main countries of import (2010-2013)	Calculated WF_{prod} of food consumed in Hong Kong (l/kg)			WF_{cons} in Hong Kong (l/cap/d)			Comments
									gn	bl	gn+bl	gn	bl	gn+bl	
								Indonesia, 49% ROW							
Cocoa Beans and products	0	18	6	11		10	1.4	100% ROW	19745	4	19749	77	0	77	Global average is taken, because HK imports cocoa beans generally through processed chocolate products from countries like China, Italy or Belgium
Tea (including mate)	0	14	3	11		11	1.5	37% China, 34% Sri Lanka, 29% ROW	9034	556	9589	37	2	39	
Alcoholic beverages															
Wine	0	47	17	31	OTHER 1	30	4.1	32% France, 15% Australia, 13% USA, 40% ROW	846	1149	1995	10	13	22	Impact predominately through grape production
Beer	62	145	45	162	OTHER 6	156	23.2	44% China, 25% Republic of Korea, 31% ROW	158	8	165	10	0	11	
Meat+offals															
Bovine Meat	9	249	62	196	OTHER 18	179	25.1	38% Brazil, 28% USA, 34% ROW	11238	283	11521	772	19	791	
Mutton & Goat Meat	0	20	2	17	OTHER 3	15	2.0	Meat sheep (90% of sheep+goat meat) 41% New Zealand, 40% Australia, 19% ROW<br>Meat goats (10% of sheep+goat meat) 91% China, 9% ROW	6214	352	6566	35	2	37	
Pigmeat	123	578	166	535	OTHER 88	446	62.6	24% China, 18% Brazil, 9% Spain, 8% USA, 8%	3658	375	4034	628	64	692	Domestically produced pigmeat as reported in the FAO FBS, is constituted for 80% of live animal

Food item	PROD (10^3 tonnes)	IMP (10^3 tonnes)	EXP (10^3 tonnes)	Domestic supply (10^3 tonnes)	FEED/PROC/OTHER (10^3 tonnes)	Food supply (10^3 tonnes)	Food supply (kg/cap/yr)	Main countries of import (2010-2013)	Calculated WF_{prod} of food consumed in Hong Kong (l/kg)			WF_{cons} in Hong Kong (l/cap/d)			Comments
									gn	bl	gn+bl	gn	bl	gn+bl	
								Germany, 33% ROW							import from mainland China (slaughtered in Hong Kong)
Poultry Meat	29	1214	755	488	OTHER 51	438	61.4	28% Brazil, 32% USA, 40% ROW	3148	191	3339	530	32	562	
Meat, Other	7	17	2	23	OTHER 1	21	3.0	77% China, 23% ROW	6015	283	6298	50	2	52	Average WF_{prod} of beef, pork and poultry is taken
Offals	12	740	497	255	OTHER 81	175	24.5	Offals cattle (35% of all offals) 65% Brazil, 35% ROW Offals pigs (63% of all offals) 25% Germany, 17% USA, 58% ROW Offals sheep (3% of all offals) 41% Australia, 40% New Zealand, 19% ROW	4688	133	4822	315	9	324	
Animal fats															
Butter, Ghee	0	13	4	8		8	1.2	32% Australia, 27% New Zealand, 41% ROW	3913	371	4284	13	1	14	
Cream	0	2	1	1		1	0.1	36% France, 64% ROW	1116	105	1221	0	0	0	
Fats, Animals, Raw	42	74	85	32	OTHER 7	36	5.4	23% Indonesia, 21% Chinese Taiwan, 56% ROW	3739	316	4055	55	5	59	
Eggs															
Eggs	0	109	1	108	OTHER 3	100	14.0	59% China, 23% USA, 18% ROW	1982	195	2177	76	7	84	
Milk (excl. Butter)															

Food item	PROD (10^3 tonnes)	IMP (10^3 tonnes)	EXP (10^3 tonnes)	Domestic supply (10^3 tonnes)	FEED/PROC/OTHER (10^3 tonnes)	Food supply (10^3 tonnes)	Food supply (kg/cap/yr)	Main countries of import (2010-2013)	Calculated WF_{prod} of food consumed in Hong Kong (l/kg)			WF_{cons} in Hong Kong (l/cap/d)			Comments
									gn	bl	gn+bl	gn	bl	gn+bl	
Milk	0	801	87	715	OTHER 35	679	95.2	23% Netherlands, 15% China, 13% Australia, 49% ROW	760	82	841	198	21	219	
Fish and seafood															
Freshwater Fish	3	32	2	33		33	5	No data	1629	5379	7008	22	71	93	100% of freshwater fish: Feed and pond evaporation assumed
Demersal, pelagic and other marine fish	154	247	166	236	FEED 22	213	32	No data	1629	179	1808	140.7	15.5	156.1	100% of all fish: Feed assumed
Crustaceans, Cephalopods, Molluscs	17	224	61	179		179	27	No data	546	60	606	39.8	4.4	44.1	100% of crustaceans: Feed assumed<br>Cephalopods and Molluscs: No feed assumed
